# Greater accumulation of litter in spruce (*Picea abies*) compared to beech (*Fagus sylvatica*) stands is not a consequence of the inherent recalcitrance of needles

**DOI:** 10.1007/s11104-012-1165-z

**Published:** 2012-03-07

**Authors:** Torsten W. Berger, Pétra Berger

**Affiliations:** Department of Forest- and Soil Sciences, Institute of Forest Ecology, University of Natural Resources and Live Sciences (BOKU), Peter Jordan-Straße 82, 1190 Vienna, Austria

**Keywords:** Decomposition, *Fagus sylvatica*, Litter quality, Litterbag, Mixing effects, *Picea abies*

## Abstract

**Background and aims:**

Replacement of beech by spruce is associated with changes in soil acidity, soil structure and humus form, which are commonly ascribed to the recalcitrance of spruce needles. It is of practical relevance to know how much beech must be admixed to pure spruce stands in order to increase litter decomposition and associated nutrient cycling. We addressed the impact of tree species mixture within forest stands and within litter on mass loss and nutritional release from litter.

**Methods:**

Litter decomposition was measured in three adjacent stands of pure spruce (*Picea abies*), mixed beech-spruce and pure beech (*Fagus sylvatica*) on three nutrient-rich sites and three nutrient-poor sites over a three-year period using the litterbag method (single species and mixed species bags).

**Results:**

Mass loss of beech litter was not higher than mass loss of spruce litter. Mass loss and nutrient release were not affected by litter mixing. Litter decay indicated non-additive patterns, since similar remaining masses under pure beech (47%) and mixed beech-spruce (48%) were significantly lower than under pure spruce stands (67%). Release of the main components of the organic substance (C_org_, N_tot_, P, S, lignin) and associated K were related to mass loss, while release of other nutrients was not related to mass loss.

**Conclusions:**

In contradiction to the widely held assumption of slow decomposition of spruce needles, we conclude that accumulation of litter in spruce stands is not caused by recalcitrance of spruce needles to decay; rather adverse environmental conditions in spruce stands retard decomposition. Mixed beech-spruce stands appear to be as effective as pure beech stands in counteracting these adverse conditions.

## Introduction

Since it became popular to plant Norway spruce (*Picea abies*) outside its climatic range to reforest devastated forest land in Central Europe in the 19th century, spruce and beech (*Fagus sylvatica*) stands have been contrasted in their effects on the forest soil. There is a large body of scientific work comparing the mineral nutrition and nutrient cycling in pure spruce and pure beech stands (Wittich 1933; Ellenberg *et al*. 1986; Kreutzer *et al*. 1986; Matzner 1988; Bücking and Steinle 1991; Ende 1991; Heitz 1998; Croise *et al*. 1999; for a good overview in German see Rehfuess 1990, pp145–170). As stated in this rather old literature, the chemically and mechanically well protected, long-living foliage of spruce decomposes slowly, resulting in the buildup of forest floor humus and consequently sequestration of nutrients in organic matter, acidification of the top soil and reduced activity of soil macro fauna. Beech, the “mother of the forests” as it is often called in popular German forest writing, is said to counteract soil degradation by faster decomposition of its litter, by recycling of nutrients from deeper soil horizons through its deeper root system, and by creating root channels, which allow deeper rooting of spruce thereby increasing the stability of the stand against wind throw. From the viewpoint of resistance against pests and diseases, mixtures of different tree species are considered more stable than monocultures. It is nowadays considered prudent, close-to-nature forest practice, to convert secondary spruce stands into mixed beech-spruce stands, even though such mixtures have often not been the natural vegetation at most of the sites in question. However, studies on the combined effects of beech-spruce mixtures are very rare, although mixed beech-spruce forests are a major forest type in Central Europe. For example, in a review by De Schrijver *et al*. (2007) the most abundant coniferous/deciduous combination is *Picea abies*/*Fagus sylvatica* (16 out of 38 studies) but only one pair included a comparison between a pure spruce and a mixed spruce-fir-beech stand.

Because patterns of properties observed in mixed stands cannot be predicted from patterns observed in monocultures (*e.g.*, Finzi and Canham 1998), the assumption that mixed beech-spruce stands are a suitable replacement for secondary spruce stands on former mixed broadleaf sites needs critical review. Binkley and Giardina (1998) concede acidification by spruce but question its negative consequences on stand growth, since aboveground net primary production is higher in spruce forests than in beech. There is no evidence that deciduous admixture improves foliar nutrition of conifers (Rothe *et al*. 2003), since soil nutrient pools are not necessarily linked with nutrient levels. The rare studies on the effect of beech-spruce mixtures (Rothe 1997; Rothe and Binkley 2001; Rothe *et al*. 2002; Berger *et al*. 2002, 2004, 2006, 2009b) do not generally justify the long-held “beech - mother of forests” concept. Studies by Schmid and Kazda (2001; 2002) do not support the common belief that beech roots “open up” the soil for deeper rooting of spruce. In these cases, the presence of beech in a spruce plantation causes the formation of a two-storey root distribution, with spruce occupying a rather shallow domain in the topsoil. Sterba *et al*. (2002) conclude that Norway spruce grows better in pure stands than in otherwise comparable mixed species stands and link this to water stress arising from competition with beech as a result of the very shallow rooting system of spruce trees in a mixed stand.

We hold up the old idea of beech as site improver of spruce stands to the harsh light of rigorous scientific testing. Previous research has focused on nutrient cycling (soil leaching, Berger *et al*. 2009a) and soil respiration (C sequestration, Berger *et al*. 2010), conducted at the same 18 forest sites of this study. Results have shown so far, this old idea may be true on occasion, it is often not, and the opposite is true on other occasions. For example, nitrate and sulfate seepage losses of mixed beech-spruce stands are higher than expected from the corresponding pure-species stands due to an unfavorable combination of high spruce-similar soil solution concentrations coupled with high beech-similar water fluxes. As a consequence the mixed beech-spruce stands on nutrient-rich soils (bedrock: Flysch) have the highest soil acidification rates (Berger *et al*. 2009a). We expected highest soil C sequestration under beech due to its deep rooting system, but on both bedrocks, contributions of beech stands to net soil C sequestration was lower than of the corresponding (mixed) spruce stands, since C losses (heterotrophic soil respiration) were relatively high compared to small C inputs (leaf and root litter; Berger *et al*. 2010). “Although we do not actively manage litter decomposition, several assumptions about decomposition are implicit in our expectations. For example, we expect that adding or increasing the broadleaf component will improve the site by increasing nutrient cycling and availability, partly through its higher quality litter and faster decay (Prescott *et al*. 2004)”. Hence, within this third publication on the same study sites we focus on purported “safe” generalizations about beech litter and its decomposition in single and mixed litter combinations.

Decomposition processes are important for cycling of nutrients in forest ecosystems and are influenced by macro- and micro-climate, litter quality, activity of decomposing organisms and soil nutrient status (Vesterdal 1999). Replacement of beech by spruce is associated with changes in soil acidity, soil structure and humus form, which are commonly ascribed to the recalcitrance (*e.g.*, high C/N ratios and lignin concentrations) of spruce (*e.g.*, Ellenberg *et al*. 1986). The formation of thick organic layers in monocultures of spruce is associated with reduced tree growth and therefore “hampers forest productivity” (Kazda and Pichler 1998). Hence, knowing how much beech must be admixed to pure spruce stands in order to increase litter decomposition, is of practical relevance for forest management strategies, since conversion of secondary pure spruce stands to mixed species stands is a current issue in Europe (Spiecker *et al*. 2004).

Mixing litter from species with differing resource quality and leaf structure changes the chemical environment and physically alters the total litter surface where decomposition is occurring (Hector *et al*. 2000). These alterations can also affect decomposer abundance and activity (Scheu *et al*. 2003). Thus, chemical and physical changes in leaf mixtures can influence decomposition rates both directly (physically) and indirectly (through the decomposer community and its activities). Gartner and Cardon (2004) found 30 papers that focus directly on decomposition of mixtures of litters, assessing whether decay rates in species mixtures can be predicted from known decay rates of the component litters (additive effects) decaying alone, but, *i.e.*, not a single paper in this review explored decomposition of mixed beech-spruce litter, simultaneously examining the decay of the component single species. The term “decomposition”, used in this study, comprises both mass loss (decay rate) and nutrient release (including nutrient transfers among leaves of different species), which are not necessarily linked with each other. The review by Gartner and Cardon (2004) revealed that nutrient transfer among leaves of different species is striking, with 76% of the mixtures showing non-additive dynamics of nutrient concentrations. In accordance with the comprehensive work of Wardle *et al*. (1997) these non-additive effects of decomposing mixed litter are difficult to generalize. Whether nutrient transfers within the decomposing litters are mediated by physical (*e.g.*, leaching) or biological (*e.g.*, fungi) means, nutrients released from rapidly decaying, higher quality litter can stimulate decay in adjacent, more recalcitrant litters (McTiernan *et al*. 1997; Sariyildiz *et al*. 2005) or conversely, leaf litter decay can be slowed by release of inhibitory compounds such as phenolics and tannins (Fyles and Fyles 1993; Prescott *et al*. 2000). There are also recent examples that decay rates of litter mixtures may display additive characteristics (Vivanco and Austin 2008; Hoorens *et al*. 2010; Jacob *et al*. (2010).

We measured litter decomposition in three adjacent stands of pure spruce (*Picea abies*), mixed beech-spruce and pure beech (*Fagus sylvatica*) on three nutrient-rich sites (bedrock: Flysch) and three nutrient-poor sites (bedrock: Molasse; yielding a total of 18 stands) over a three-year period using the litterbag method to evaluate our working hypothesis: Decomposition and nutrient release of foliage litter of beech and spruce is a function of litter quality and incubation site, indicating non-additive effects of litter mixtures. We addressed the impact of tree species composition within forest stands and litter by asking the following related questions:

1. Does beech litter decompose faster than spruce litter?
2. Does litter decompose faster in beech or beech-spruce forests than in spruce forests?
3. Does mixing of beech and spruce litter hasten decomposition of spruce litter?
4. Does mass loss (decay rate) correlate with nutrient release?
5. Which parameters (litter, soil, environment) represent the best suite of characteristics that actually control decay rates and nutrient release?

## Materials and methods

### Study sites

Six sites were selected on the two different bedrocks Flysch and Molasse (3 comparable sites on each substrate). Beech and spruce were similarly mixed, before one stand at each site was converted into the current pure spruce stand. According to Rothe and Binkley (2001) research could take advantage of the spatial scale at which trees interact in the absences of replicated-plot experiments. Hence, for this study we selected mono specific beech stands (5–7 canopy dominant trees) within the mixed species stands. Individual trees influence soil properties primarily within the radius of the canopy (*e.g.*, review by Rhoades 1997). The current design with 3 tree species compositions (spruce, mixed, beech) per site and 3 site replicates per 2 bedrocks (total of 18 stands) enabled testing mixed species effects via the factor incubation site. Nutrient fluxes had been monitored by us (Berger *et al*. 2009a) for the same 18 stands. Detailed site information is given by these authors for each of the 18 stands. Mean stand characteristics are listed in Table 1. Standing timber volume and dominant tree heights are higher on Flysch, despite a somewhat younger stand age. On average, the stands are located on N (Flysch) to W (Molasse) facing slopes. Precipitation declined from the western (Molasse) to the eastern (Flysch) parts of Austria.

**Table 1:** Characteristics of adjacent pure and mixed species stands of spruce and beech at the experimental sites at Flysch and Molasse according to a 1997 survey (3 sites per bedrock; each value is the mean of 3 stands). Mono specific beech stands (5–7 canopy dominant trees) were selected within the mixed species stands. Hence, ha-related stand characteristics are the same for the mixed and the adjacent pure beech stand, except for one site on Flysch, where the pure beech stand was large enough. Individual characteristics of all 18 stands are given by Berger *et al*. (2009a)

Incubation stand	Age	Stems	Timber volume	Basal area	Dominant tree height	Elevation	Slope	Aspect (from N to E)	Precipitation (1971-2000)	N coordinates		E coordinates	
	years	N ha^-1^	m^3^ ha^-1^	m^2^ ha^-1^	m	m a.s.l.	degrees	degrees	mm year^-1^	range		range	
FLYSCH													
Spruce	68	750	633	65	32.7	590	14	352.5	1043	47°56′25″	48°05′50″	14°11′12″	15°39′46″
Mixed	76	572	632	44	34.3	587	13	352.5	1043	47°56′21″	48°05′50″	14°12′43″	15°39′49″
Beech	76	567	666	45	35.0	587	13	352.5	1043	47°56′21″	48°05′50″	14°12′44″	15°39′54″
MOLASSE													
Spruce	79	803	430	49	27.7	640	9	262.5	1180	48°05′10″	48°05′27″	13°14′08″	13°18′36″
Mixed	91	391	354	37	29.7	650	12	255.0	1180	48°05′10″	48°05′33″	13°14′14″	13°18′39″
Beech	91	391	354	37	29.0	647	11	285.0	1180	48°05′11″	48°05′35″	13°14′14″	13°18′36″

The following site descriptions, given by Berger *et al*. (2009a), bear repeating at this point. The study sites on Flysch are spread throughout Lower and Upper Austria at elevations between 480 and 730 m (Table 1). The Flysch zone is a narrow strip in the foothills of the Northern Limestone Alps from west to east throughout the country. Flysch consists mainly of old tertiary and mesozoic sandstones and clayey marls. Nutrient release from this bedrock is high and consequently the prevalent humus forms are mull (beech and mixed stands) to intermediate types between mull and moder (pure spruce stand), indicating quick turnover of the forest litter layer (usually less than 3 cm thickness). Soil parameters (Table 2) indicate nutrient rich soils. All soils of these study sites were classified as pseudogley (Scheffer and Schachtschabel 1998; FAO classification: stagnic cambisol), since horizons with a high fraction of fine material (loam to clay) cause temporary waterlogging (stagnation zone at approximately 40–50 cm soil depth). There are hardly any shrubs and the total cover of the herb layer is between 5% (spruce) and 20% (beech). The natural forest vegetation of the mixed stands on Flysch is *Asperulo odoratae-Fagetum* (Mucina *et al*. 1993).

**Table 2:** Properties of the forest floor, 0–10 cm mineral soil and top soil (forest floor + 0–10 cm mineral soil) under the pure and mixed incubation stands of spruce and beech on the bedrocks Flysch and Molasse (each value is the mean of 3 stands), modified from Berger *et al*. (2010): total stores of C_org_ (kg m^−2^ per horizon) and C_mic_, N_tot_, P and S (g m^−2^ per horizon); stores of Ca, Mg, K, Na, Al, Fe and Mn, given as total content in the forest floor and exchangeable content in the mineral soil (g m^−2^ per horizon); cation exchange capacity (CEC), sum of acid cations (Al, Fe, Mn, H^+^) in mol_c_ m^−2^ per horizon; base saturation (%); C_org_/N_tot_ and C_org_/P ratio; C_mic_ in percent of C_org_

Incubation stand	C_org_	C_mic_	N_tot_	P	S	Ca	Mg	K	Na	Al	Fe	Mn	CEC	Acid cat.	Base sat.	C_org_/N_tot_ ratio	C_org_/P ratio	C_mic_/C_org_ (%)
FLYSCH																		
Forest floor																		
Spruce	0.98	11	39	2.8	5.1	17.0	23.0 *b*	30.5 *b*	2.0	118.5 *b*	98.9 *b*	5.7	22.3 *b*	18.7 *b*	16.3 *a*	25.4	352.3	1.2
Mixed	0.69	6	29	1.4	3.1	17.6	10.5 *ab*	14.2 *ab*	1.3	57.2 *ab*	43.4 *ab*	2.4	10.9 *ab*	8.8 *ab*	25.6 *ab*	30.7	654.8	1.0
Beech	0.39	5	12	0.7	1.3	16.8	4.3 *a*	5.7 *a*	0.4	18.3 *a*	13.3 *a*	1.6	4.2 *a*	2.8 *a*	32.7 *b*	35.5	656.6	1.3
0–10 cm																		
Spruce	2.67 *b*	32	170	24.2	26.0	29.7	2.8	12.9	7.2	58.5	0.0	2.2	9.0	6.6 *b*	23.7	17.0	111.9	1.2 *a*
Mixed	2.26 *ab*	41	120	22.2	22.1	41.8	3.4	11.8	10.3	51.0	0.0	1.3	8.9	5.7 *ab*	34.1	19.2	107.6	1.8 *ab*
Beech	2.03	42	123	24.8	22.3	55.5	4.9	18.9	9.6	33.3	0.0	1.0	7.8	3.7 *a*	50.2	19.9	84.5	2.0 *b*
Top soil																		
Spruce	3.65	44	208	27.0	31.0	46.7	25.8 *b*	43.4	9.3	177.1 *b*	98.9 *b*	7.9	31.3 *b*	25.3 *b*	19.3	18.4	136.7	1.2
Mixed	2.94	48	148	23.6	25.2	59.4	14.0 *ab*	26.0	11.6	108.2 *ab*	43.4 *ab*	3.7	19.8 *ab*	14.5 *ab*	32.1	20.3	130.4	1.7
Beech	2.42	47	135	25.5	23.6	72.3	9.2 *a*	24.6	10.0	51.6 *a*	13.3 *a*	2.6	12.0 *a*	6.6 *a*	44.7	20.6	98.0	1.9
MOLASSE																		
Forest floor																		
Spruce	5.17 *b*	37	206	9.2	26.7	35.7	26.2	34.0	6.5	245.7	239.4	3.2	45.4	40.3	11.3	25.3 *b*	569.8	0.7
Mixed	4.70 *b*	40	187	8.8	23.4	36.0	32.8	47.4	6.4	300.0	245.4	3.6	52.7	46.7	11.7	25.2 *b*	537.4	0.9
Beech	3.71 *a*	32	172	7.8	20.5	33.7	29.4	42.4	5.0	235.1	187.7	3.3	41.8	36.4	13.2	21.6 *a*	481.9	0.8
0–10 cm																		
Spruce	3.72	36	161	21.9	28.6	1.4	0.5	4.9	4.6	40.5	0.0	0.0	5.0	4.5	9.1	23.3	175.8	1.0
Mixed	3.23	41	148	22.5	26.6	0.7	0.6	5.7	4.2	43.7	0.2	0.1	5.3	4.9	8.1	23.2	147.6	1.3
Beech	4.64	55	184	32.7	41.1	1.7	1.0	9.8	6.5	58.8	0.1	0.0	7.3	6.6	9.6	24.4	145.3	1.2
Top soil																		
Spruce	8.90	73	366	31.1	55.3	37.1	26.7	38.8	11.1	286.2	239.4	3.2	50.4	44.9	11.1	24.4	292.9	0.8
Mixed	7.93	82	335	31.3	50.0	36.7	33.4	53.1	10.6	343.7	245.6	3.6	58.0	51.6	11.2	23.8	259.2	1.0
Beech	8.35	87	356	40.5	61.5	35.4	30.4	52.2	11.6	293.9	187.8	3.4	49.0	42.9	12.6	23.2	214.0	1.0
FACTOR BEDROCK (Top soil)																		
Flysch (All)	3.00 ***	46 ***	164 ***	25.4 *	26.6 ***	59.5 ^(^*^)^	16.3 **	31.3 *	10.3 ns	112.3 ***	51.9 ***	4.7 ns	21.0 ***	15.5 ***	32.0 **	19.8 ^(^*^)^	121.7 ***	1.6 **
Molasse (All)	8.39	81	352	34.3	55.6	36.4	30.2	48.0	11.1	307.9	224.3	3.4	52.5	46.5	11.7	23.8	255.4	1.0

A one-way ANOVA (factor incubation stand) was performed for each bedrock and horizon separately and results of a Duncan multiple range test are given only, if differences were significant (different letters indicate significant differences, *p* < 0.05; *a* represents the lowest mean). Another one-way ANOVA (factor bedrock) was done to test mean differences between Flysch and Molasse for the top soil; level of significance is shown as: ns: not significant, *p* > 0.10; ^(^*^)^: *p* < 0.10; *: *p* < 0.05; **: *p* < 0.01; ***: *p* < 0.001

All study sites on Molasse are located in Upper Austria, in a forested landscape, called Kobernausserwald, at elevations between 570 and 710 m (Table 1). Parent material for soil formation are tertiary sediments (so-called “Hausruck-Kobernausserwald” gravel), which consist mainly of quartz and other siliceous material (granite, gneiss, hornblende schist, pseudotachylite and colored sandstone). Because of this acidic bedrock with low rates of nutrient release, the dominant soil types are mainly semi-podzols (Scheffer and Schachtschabel 1998; intermediate soil type between cambisol and podzol; FAO classification: dystric cambisol) and partly podzols. Humus form is acidic moder and the thickness of the forest litter layer varies between 5 and 10 cm, indicating slow turnover and accumulation of nutrients. In general, soils on Molasse contain more organic carbon and are more acidic, more sandy and less supplied with nutrients than soils on Flysch (Table 2). There are no shrubs and the total cover of the herb layer is 10% (spruce) to 15% (beech). The natural forest vegetation of the mixed stands is *Luzulo nemorosae-Fagetum* (Mucina *et al*. 1993).

### Soils

We measured soil parameters of the 18 stands within a related study on soil respiration (see Berger *et al*. 2010). Forest floor (O-horizon: O_i_ + O_e_ + O_a_) and mineral soil (0–10 cm) were taken with a core sampler of 70 mm diameter in summer 2006. There were three distributed replicate samples at each stand, which were pooled before analysis. Samples of forest floor and of mineral soil (fine soil, separated by sieving <2 mm) were analyzed for total content of C (LECO SC 444, USA), N (Kjeldahl method according to ÖNORM L1082; 2300 Kjeltec Analyzer Unit, Tecator, Sweden), P and S (both after digestion with HNO_3_/HClO_4_ according to ÖNORM L1085; ICPS, inductive coupled plasma spectrometry, Optima 3000 XL, Perkin Elmer, USA). Organic carbon (C_org_) was calculated as total carbon minus CCaCO_3_ (Scheibler method: reaction of carbonates with HCl and volumetric determination of emerging CO_2_ according to ÖNORM L1084). Calcium, Mg, K, Na, Al, Fe and Mn were measured as total contents after digestion with HNO_3_/HClO_4_ in the forest floor and as exchangeable cations (0.1 M BaCl_2_ extract) in the mineral soil by ICPS. Soil acidity was measured as pH with a glass Ag/AgCl combination electrode with KCl reference electrode (10 g soil were mixed with 25 ml of 0.01 M CaCl_2_ or deionized H_2_O, stirred, and the pH was measured next morning 30 min after stirring again). Elemental stocks were then calculated as the product of dry (105°C) fine soil masses (related to area and soil depth) and corresponding element contents. Microbial C (C_mic_) was calculated as the differences in organic C between fumigated and non-fumigated (control) samples according to Schinner *et al*. (1996). Two replicates of each sample, 2.5 g fresh forest floor or 5 g fresh mineral soil, were fumigated for 24 h with ethanol-free chloroform at 25°C. Subsequently the chloroform was removed by evacuation. Fumigated samples and controls were extracted with 25 ml 0.5 M K_2_SO_4_ and filtered; extracts were kept frozen until analysis. Total dissolved organic carbon was analyzed in the extracts with a Shimadzu TOC-5050 Total Carbon Analyzer, Japan. Non-extracted amounts of microbial C were compensated for by a correction factor of *k*_EC_ = 0.35.

Volumetric soil water content was measured around 1 April, 1 June, 16 July and 1 Sept in summer 2006 (second year) and 2007 (third year of this litterbag study) with Trase TDR-systems from Soilmoisture Equipment Corp. using fixed waveguides (3 replications per stand), buried at 0–10 cm depth in the mineral soil (see Berger *et al*. 2010).

### Litterbag experiment

Fresh litter of beech and spruce was collected by spreading nets from early October to mid November 2004 under the pure stands of beech and spruce. Collected foliage litter was dried at 50°C for 48 h, however, all data given in this paper are related to 105°C dry weight, estimated from subsamples not used for the decomposition study.

Litterbags were prepared by folding strips of polyethlylene nets (1 mm mesh size) to obtain double layered bags, which were closed on the two open sides with high carbon steel paper-clips. The litterbags were filled with 3 different mixtures from the corresponding site, yielding 4 compounds to be analyzed: single spruce, SP; 1:1 mixture of spruce and beech, mSP, mBE; single beech, BE. The single spruce bags were filled with 2 g (10 × 8 cm size) and the mixed and single beech bags with 3 g (10 × 10 cm size) of dried (50°C) litter. On an area basis these litter amounts (244–293 g m^−2^; related to 105°C) represent the lower range of annual litter input (370–560 g m^−2^ year^−1^ on one of the same sites on Flysch; 310–370 g m^−2^ year^−1^ on one of the same sites on Molasse; Berger *et al*. 2009b).

In early December 2004 the litterbags were placed on the forest floor (after stripping off part of the non decayed leaves and needles of the O_i_-layer and covering the bags thereafter again) in a randomized block design with four 0.5 × 0.5 m blocks per stand. Each of the blocks contained four sets of the three litter mixtures for sampling at four different dates. The bags of each set were connected with each other by a string, tied to one wooden stick above and below each block. In addition, each individual bag was fastened to the forest floor by one 10 cm long pin of high carbon steel on the left and right side, outside the clipped seam. A total of 864 litterbags were used for the entire study (2 bedrocks × 3 sites per bedrock × 3 incubation stands × 3 litter mixtures × 4 replications per stand × 4 sampling dates =864). Litter bags were collected twice during the first year (May and November) and once during the following years (November) for a total period of 3 years. One set of bags per stand and sampling date, always retrieved from the same block, was brought in a cooling box to the laboratory and put immediately into the freezer until the measurement of C_mic_ was performed on these fresh samples. The remaining 3 sets were returned horizontally in flat, piled-up boxes to avoid mass loss via transport. After drying at 40°C the bags were opened, non-foliage litter material was sorted out and the mixed bags were separated into its components by hand. Thereafter, the components of each individual bag were dried at 105°C for 48 h, weighed and the 3 block replicates were subsequently pooled to give one sample and were ground for chemical analysis.

Initial litter contents and contents after 0.5, 1, 2 and 3 years in the pooled litter samples were analyzed for C_org_, N_tot_, P, S, Ca, Mg, K, Na, Al, Fe and Mn as described for the soil (forest floor) samples above. Total lignin content (acid-insoluble lignin plus acid-soluble lignin) was measured by Fourier transform near infrared (FT-NIR) spectrometry (Bruker FT-IR spectrometer, EQUINOX 55, Germany, equipped with NIR fibre optic (measuring the diffuse reflected light) and a germanium-diode detector, limited by a cut-off wavenumber of 5100 cm^−1^ (details of the method are given in Schwanninger *et al*. 2009). This indirect method proved to be a powerful tool for rapid estimation of the lignin content in agreement with direct classical wet-lab chemistry data (Schwanninger and Hinterstoisser 2002). Microbial C (C_mic_) was measured in the individual remaining sets as done for the fresh forest floor samples, however, only one replicate of fresh litter (1 g or sometimes less for fumigation, 1 g for the control) could be used and the dry weight for the conversion factor fresh/dry had to be retrieved from the corresponding pooled samples because of small sample volumes.

### Data evaluation and statistics

Mass loss was calculated as the difference between the initial dry mass (M_o_) and the actual dry mass (M_t_) at each sampling date. Mass loss over time (t; years) was approximated using the standard single exponential decay function: ${ \ln }\left( {{{\text{M}}_{\text{t}}}/{{\text{M}}_{\text{o}}}} \right) = - k{\text{t}}$, where *k* is the decomposition rate (year^−1^). Linear regressions were performed setting the intercept to zero (Vivanco and Austin 2008). Nutrient release was estimated initial content minus content at each sampling date and expressed either in % of the initial content or in mg g^−1^ incubated litter.

One-way ANOVAs were performed for each bedrock (soil type) separately to test whether significant differences of soil properties (incubation stand) and initial litter chemistry (litter mixture) were caused by the corresponding grouping variables (given in parentheses). Additional one-way ANOVAs (factor bedrock) were performed to test differences between the two bedrocks Flysch and Molasse. However, net nutrient release was regressed against initial nutrient contents for each incubation stand (grouped by litter mixture) over both bedrocks (soil type), in case soil type indirectly effects litter quality, increasing the range of the data.

The largest data set was used for the performance of a four-way (2 × 3 × 4 × 4) ANOVA to test effects of bedrock (nutrient rich soils on Flysch *versus* nutrient poor soils on Molasse), incubation stand (spruce, mixed, beech), litter mixture (single needles, mixed needles, single leaves, mixed leaves) and sampling date (after 0.5,1, 2 and 3 years) on the remaining mass, element contents (percentage of initial values) and selected compound ratios of litter enclosed in the litter bags (*N* = 2 bedrocks × 3 incubation stands × 3 replications; sites × 4 litter mixtures × 1 stand mean of three replicated litter bags × 4 sampling dates =288). For the decomposition constant *k* a three-way ANOVA without the factor sampling date was done, since one *k*-value was deduced over the whole 3-years period. In case of significant interactions between the grouping factors these factors can not be tested individually but affect the dependant factor jointly. Finally, the same parameters were calculated for the grouping variables bedrock, incubation stand and litter mixture (2 × 3 × 4 ANOVA) after 3 years of decomposition and differences between spruce, mixed and beech stands (incubation stand) as well as between SP, mSP, mBE and BE (litter mixture) were compared by Duncan multiple range tests.

To address question 5, which parameters (litter, soil, environment) represent the best suite of characteristics that actually control decay rates and nutrient release, we first performed bivariate correlations between *k* (year^−1^) and net nutrient release (mg g^−1^ litter) of exposed spruce and beech litter in single (SP and BE) and mixed (mSP and mBE) bags over 3 years, respectively, and initial nutrient contents of litter (including selected element ratios of litter) and soil parameters (separated by horizons; *N* = 2 bedrocks x 3 incubation stands × 3 replications; sites × 2 litter mixtures × 1 stand mean of three replicated litter bags =36). Admixture of beech represented an environmental parameter, which was added as dummy variable (0= spruce stand, 50= mixed stand, 100= beech stand). This variable may be important if features of the soil environment (*e.g.*, micro-climate, physical conditions, activity of decomposing organisms), not encompassed by the manifold soil chemical parameters, are primarily driving decomposition. Assumedly, effects of tree species composition within forest stands are expressed via litter quality and soil parameters (plant-soil feedback). Hence, comparing two suites of characteristics separately for spruce and beech litter in single-species and mixed litter bags justifies conclusions about litter mixture effects, since all measured parameters in litter, soil and environment were identical.

In a second step, those pre-selected parameters which correlated significantly with *k* or net release of the individual nutrients were used to run stepwise regressions to find the driving forces (independent variables) of *k* and release (at each step, the independent variable not in the equation that has the smallest probability of *F* is entered, if that probability is sufficiently small; the method terminates when no more variables are eligible for inclusion or removal). Stepwise regression is a method of data reduction, taking inter-correlations into account. For that reason we excluded *k* as independent variable, since we were interested in the best suite of individual parameters (and processes) that control decay rates, while the decomposition constant *k per se* integrates most (unknown) controlling factors. All statistics were performed with the package PASW Statistics 17 (Release 17.0.2, 11 March 2009).

## Results

### Soils

Soil properties of the top soil (forest floor + 0–10 cm mineral soil) indicated significant differences between the soils on Flysch and Molasse for all listed parameters (Table 2) except stores of Na and Mn. On Molasse, forest floor and consequently top soil contents were significantly higher for all element stores except Ca. Flysch sites had higher base saturation, higher C_mic_/C_org_ ratios but lower C_org_/N_tot_ and C_org_/P ratios of the top soil. Comparing individual base cation storages within the 0–10 cm mineral soil, justifies calling soils on Flysch nutrient-rich and soils on Molasse nutrient-poor.

Effects of species composition (incubation stand) were much more pronounced on soils formed over Flysch than on Molasse as documented elsewhere (Berger *et al*. 2002; 2004). On Flysch, spruce stands had significantly higher stores of C_org_, Al, Fe and sum of acid cations and a lower base saturation (Table 2). Mean pH (H_2_O) at 0–10 cm increased from 4.3 (spruce) to 4.7 (mixed) to 5.2 (beech; beech > spruce; mixed = spruce, beech; not shown in Table 2). The C_org_/N_tot_ and C_org_/P ratios in the forest floor on Flysch tended to increase from spruce to mixed to beech stands. Recalcitrant components of the non-foliage fraction with disproportionally wider C_org_/N_tot_ and C_org_/P ratios (branches, fruit capsules) dominate the beech litter at Flysch. Spruce sequestered more C_org_, Mg and K (higher CEC) in the forest floor than beech on Flysch. On Molasse, the C_org_/N_tot_ ratio in the forest floor declined from spruce to beech (beech < mixed, spruce; the C_org_/P ratio showed a declining trend as well) since C_org_ stores showed a similar pattern (beech < mixed, spruce). No other significant effects of tree species were visible on Molasse. Mean pH (H_2_0) at 0–10 cm was the same in all stand compositions (4.1).

### Initial litter quality

Initial element contents and ratios of lignin/N_tot_, C_org_/N_tot_, C_org_/P and C_mic_/C_org_ of spruce (SP) and beech (BE) litter, collected at adjacent spruce and beech stands on Flysch and Molasse in fall 2004, are given in Table 3. Base cation contents (Ca, Mg and K) were higher in beech than in spruce. However, in all other cases differences were negligible or indicated even lower quality of beech litter (except for the C_mic_/C_org_ ratio): lower N_tot_ contents coupled with higher C_org_ contents, higher lignin/N_tot_, C_org_/N_tot_ and C_org_/P ratios for beech than for spruce. Comparisons between beech and spruce foliage at the same 6 mixed beech-spruce stands by Berger *et al*. (2009a) indicated significantly higher nutrient concentrations of beech foliage for all elements, except Mn (both substrates) and P (Flysch). Hence, in accordance to Kristensen *et al*. (2004) and Berger *et al*. (2009b) retranslocation of nutrients prior to senescence is a more important process in beech than in spruce foliage, minimizing or turning around differences of associated litter quality. The quite different nutritional status between soils on Molasse and on Flysch (Table 2) was hardly reflected in initial litter chemistry, except for C_org_, N_tot_, Ca and Mg. Litter lignin contents were significantly higher on Molasse than on Flysch.

**Table 3:** Initial nutrient contents (mg g^-1^), ratios of lignin/N_tot_, C_org_/N_tot_ and C_org_/P and C_mic_ in percent of C_org_ of spruce (SP) and beech (BE) litter, collected at adjacent spruce and beech stands on Flysch and Molasse (3 sites per bedrock; each value is the mean of 3 stands) in fall 2004

Litter mixture	C_org_	C_mic_	N_tot_	P	S	Ca	Mg	K	Na	Al	Fe	Mn	Lignin	Lignin/N_tot_ ratio	C_org_/N_tot_ ratio	C_org_/P ratio	C_mic_/C_org_ (%)
FLYSCH																	
SP	488.7	0.9	9.4	0.6	1.0	10.7 **	0.7 *	1.9	0.1	0.9	0.7	1.5	365.9	40.1	54.9	899.6	0.2
BE	498.0	4.7	8.6	0.4	1.1	17.6	1.4	2.5	0.2	0.1	0.2	0.9	414.8	49.2	58.9	1650.6	0.9
MOLASSE																	
SP	504.5 *	0.8	15.4 ^(^*^)^	0.9 **	1.2	3.1	0.5 *	1.1 **	0.1	0.5 ^(^*^)^	0.4	0.4	419.2 ^(^*^)^	27.9 **	34.3 ^(^*^)^	601.9 *	0.2
BE	515.9	7.3	10.1	0.4	1.0	3.8	0.7	2.5	0.2	0.1	0.2	0.7	495.3	49.7	52.0	1425.3	1.4
FACTOR BEDROCK																	
Flysch (All)	493.3 **	2.8	9.0 ^(^*^)^	0.5	1.1	14.2 ***	1.0 *	2.2	0.1	0.5	0.4	1.2 *	390.3 *	44.6	56.9	1275.1	0.6
Molasse (All)	510.2	4.1	12.7	0.6	1.1	3.5	0.6	1.8	0.2	0.3	0.3	0.6	457.3	38.8	43.2	1013.6	0.8

A one-way ANOVA (factor litter mixture) was performed to test initial chemical differences for each bedrock separately (*N* = 3 sites x 2 litter mixtures =6). Mean differences between Flysch and Molasse were tested for all litter mixtures by another one-way ANOVA (*N* = 2 bedrocks x 3 sites x 2 litter mixtures =12); only significant results are shown as: ^(^*^)^: *p* < 0.10; *: *p* < 0.05; **: *p* < 0.01; ***: *p* < 0.001

### Mass loss

**Fig. 1:**
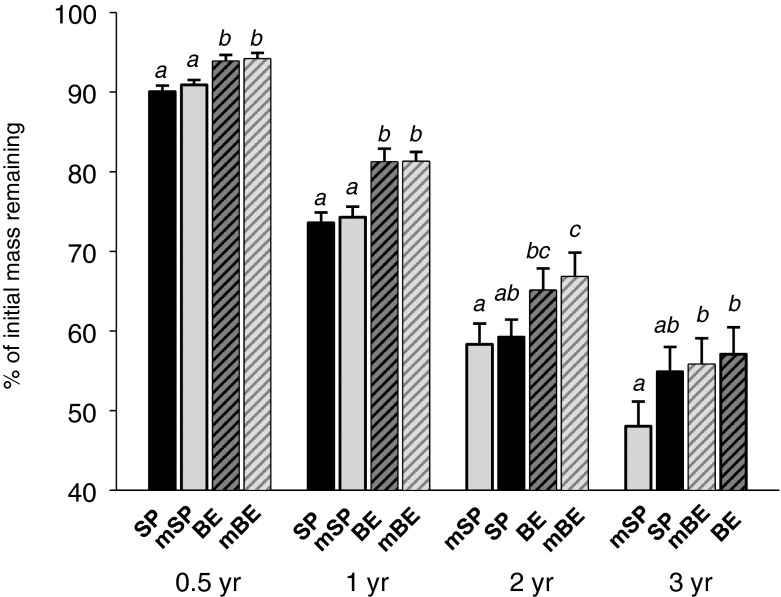
Remaining mass (%) of exposed litter mixtures in single spruce (SP), mixed (mixed spruce, mSP; mixed beech, mBE) and single beech (BE) litter bags. Since bedrock did not affect remaining mass (see Table 4) a two-way ANOVA (factors incubation stand and litter mixture) was performed for each sampling date after 0.5, 1, 2 and 3 years. Plotted bars represent group means of each litter mixture (standard errors were calculated for *N* = 2 bedrocks ×3 incubation stands ×3 replications; sites =18) and different letters indicate significant differences between them (Duncan multiple range test, *p* < 0.05)

As expected, the remaining mass of incubated litter was primarily affected by the time of exposure (sampling date; Table 4). Additionally, mass loss (100 - remaining mass in %) was significantly affected both by incubation stand and to a minor extent by litter mixture according to given *F*-values. Surprisingly, the soil type (bedrock) did not influence decay at all. It is striking that these significant differences between the 4 individual litter components (litter mixture) did not vary with tree stand composition (incubation stand), since there was no interaction between these two factors. For that reason, the remaining masses of the individual litter mixtures (SP, mSP, mBE, Be) were plotted for each sampling date, averaged over bedrock and incubation stand (Fig. 1). For each sampling date mass loss did not differ between single and mixed spruce litter or between single and mixed beech litter, however beech decomposed slower than spruce. During the first two years admixed spruce needles tended to slow down decomposition of beech foliage while mixing both fractions increased decay of spruce needles after the first year.

**Table 4:** ANOVA table of *F*-values on the effects of bedrock (nutrient rich soils on Flysch *versus* nutrient poor soils on Molasse), incubation stand (spruce, mixed, beech), litter mixture (single needles, mixed needles, single leaves, mixed leaves) and sampling date (after 0.5, 1, 2 and 3 years) on the decomposition rate (*k*), remaining mass and element contents (percentage of initial values) and selected compound ratios of litter enclosed in mesh bags

Parameter	Bedrock (B)	Incubation stand (I)	Litter mixture (L)	Sampling date (S)	Significant interactions
*k*	0.0	22.2 ***	2.9 *	–	
Mass	0.0	56.5 ***	12.6 ***	318.5 ***	I x S***
C_org_	6.2 *	69.4 ***	4.8 **	473.0 ***	I x S***
C_mic_	2.6	1.3	7.4 ***	9.5 ***	B x S***, l x S**
N_tot_	44.3 ***	28.9 ***	61.7 ***	8.5 ***	I x S***
P	4.4 *	11.7 ***	111.1 ***	1.1	I x S**, l x S***
S	52.1 ***	44.2 ***	68.2 ***	47.1 ***	B x L***, B x S**, I x S***, L x S***
Ca	7.1 **	1.4	6.7 ***	8.1 ***	B x L***, B x S***, L x S***, B x L x S***
Mg	0.6	1.6	15.1 ***	122.5 ***	B x I**, B x L***, L x S***
K	0.2	2.0	3.8 *	144.9 ***	B x I**, B x L***, B x S***, I x S***, L x S***
Na	0.1	0.4	5.9 **	1.5	
Al	55.6 ***	2.2	21.8 ***	14.9 ***	B x L*, B x S**, L x S*
Fe	40.6 ***	4.7 *	7.4 ***	13.7 ***	B x S*
Mn	2.0	1.1	5.5 **	3.5 *	B x L***, B x S***, L x S***
Lignin	72.2 ***	33.6 ***	1.5	236.3 ***	B x S**, I x S***
Lignin/N_tot_	100.5 ***	0.8	9.5 ***	142.4 ***	B x L***, B x S*, B x L x S*
C_org_/N_tot_	55.2 ***	1.0	2.4	166.4 ***	B x L***, B x S*
C_org_/P	18.5 ***	0.4	10.6 ***	39.4 ***	L x S***
C_mic_/C_org_ (%)	5.9 *	1.1	0.6	24.6 ***	B x I**, B x S***, I x S**, L x S***

A four-way (2 × 3 × 4 × 4) ANOVA was performed for each parameter (*N* = 2 bedrocks x 3 incubation stands x 3 replications; sites x 4 litter mixtures x 1 stand mean of three replicated litter bags x 4 sampling dates =288), except for *k* a three-way ANOVA without the factor sampling date was done, since one *k*-value was deduced over the whole 3-years period. Significant interactions between the grouping factors indicate that these factors can not be tested individually but affect the dependent factor jointly. Only significant results are shown as: *: *p* < 0.05; **: *p* < 0.01; ***: *p* < 0.001

**Fig. 2:**
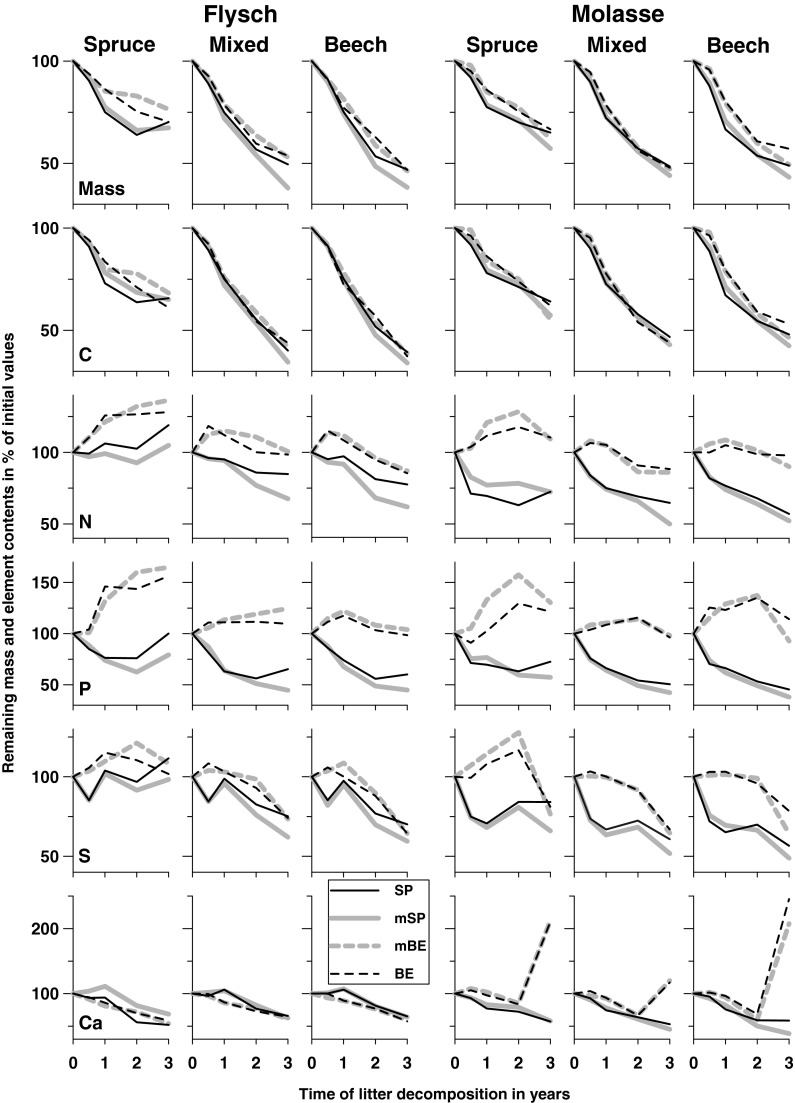
Mean remaining mass and contents of C_org_, N_tot_, P, S and Ca (percent of initial values) of exposed litter mixtures in single spruce (SP), mixed (mixed spruce, mSP; mixed beech, mBE) and single beech (BE) litter bags after 0.5, 1, 2 and 3 years, grouped by bedrock (Flysch, Molasse) and incubation stand (spruce, mixed, beech)

The only interaction (four-way ANOVA; Table 4) was between incubation stand and sampling date, indicating that the temporal pattern of remaining mass was influenced by tree stand composition, plotted in Fig. 2 for each bedrock separately. Finally, the average remaining mass after three years of decomposition amounted to 67% (spruce), 48% (mixed) and 47% (beech; spruce > mixed = beech; Table 5). The associated mass losses corresponded to *k*-values of 0.157 (spruce), 0.273 (mixed) and 0.282 (beech; spruce < mixed = beech; Table 5) and the decomposition rate was exactly the same for both soil types (bedrocks; 0.237; data in year^−1^). Hence, *k* was mainly controlled by incubation stand and the factor litter mixture (*k*-values between 0.214 and 0.276) was significant but of minor importance. There were no interactions between the 3 factors bedrock, incubation stand and litter mixture (Table 4).

**Table 5:** Mean decomposition rate (*k*; year^-1^), remaining mass and element contents (% of initial values) and selected compound ratios of litter after 3 years of decomposition for the grouping factors bedrock (soil type), incubation stand (tree species composition) and litter mixture (litter species composition: single spruce, SP; mixed spruce, mSP; mixed beech, mBE; single beech, BE)

Parameter	Bedrock		Incubation stand			Litter mixture			
	Flysch	Molasse	Spruce	Mixed	Beech	SP	mSP	mBE	BE
*k*	0.237	0.237	0.157 *a*	0.273 *b*	0.282 *b*	0.243 *ab*	0.276 *b*	0.215 *a*	0.214 *a*
Mass	54.7	53.1	66.9 *b*	47.7 *a*	47.1 *a*	54.9 *ab*	48.0 *a*	55.8 *ab*	57.1 *b*
C_org_	47.5	50.5	62.4 *b*	42.2 *a*	42.4 *a*	50.7	46.1	49.1	50.2
C_mic_	141.0	326.0	323.8	274.1	132.3	450.0 *b*	534.7 *b*	46.3 *a*	37.9 *a*
N_tot_	96.0	80.6 **	108.8 *b*	80.1 *a*	76.1 *a*	82.1 *a*	68.2 *a*	101.6 *b*	101.4 *b*
P	96.0	79.9 *	110.2 *b*	78.9 *a*	74.7 *a*	65.6 *a*	51.0 *a*	119.1 *b*	116.0 *b*
S	80.2	66.6 ***	90.9 *b*	66.1 *a*	63.3 *a*	76.4 *b*	64.4 *a*	75.2 *b*	77.7 *b*
Ca	61.0	118.6 ***	96.3	73.6	99.5	58.5 *a*	56.1 *a*	119.0 *b*	125.5 *b*
Mg	60.7	61.6	64.9	55.4	63.2	59.8	53.9	64.8	66.2
K	34.0	27.5 *	36.8 *b*	27.4 *a*	28.0 *a*	36.4 *b*	26.0 *a*	30.9 *ab*	29.8 *ab*
Na	154.2	141.7	172.4	150.2	121.3	216.8 *b*	214.4 *b*	76.6 *a*	84.0 *a*
Al	932.5	352.1 **	734.3	592.9	599.7	390.9 *a*	166.2 *a*	995.2 *b*	1016.8 *b*
Fe	722.7	289.9 **	678.4	424.9	415.6	515.1 *ab*	221.6 *a*	549.1 *ab*	739.4 *b*
Mn	87.4	129.1 *	120.1	89.2	115.3	78.8 *a*	70.9 *a*	141.8 *b*	141.5 *b*
Lignin	61.4	45.7 ***	66.6 *b*	47.6 *a*	46.5 *a*	59.5 *b*	50.1 *a*	51.6 *ab*	52.9 *ab*
Lignin/N_tot_	28.6	21.6 ***	25.2	24.6	25.6	24.9	24.9	24.9	25.8
C_org_/N_tot_	28.1	26.7	28.5 *b*	26.3 *a*	27.5 *ab*	26.7 *a*	29.1 *b*	26.6 *a*	27.3 *ab*
C_org_/P	638.0	625.2	634.3	616.7	643.7	572.1 *a*	665.5 *b*	628.0 *ab*	660.6 *b*
C_mic_/C_org_ (%)	0.4	1.1 **	0.8 *ab*	1.1 *b*	0.5 *a*	0.9 *ab*	1.3 *b*	0.6 *a*	0.5 *a*

A three-way (2 × 3 × 4) ANOVA was performed for each parameter (*N* = 2 bedrocks x 3 incubation stands x 3 replications; sites x 4 litter mixtures x 1 stand mean of three replicated litter bags = 72). Only significant differences between Flysch and Molasse (factor bedrock) are shown as: *: *p* < 0.05; **: *p* < 0.01; ***: *p* < 0.001. Significant results of a Duncan multiple range test are given for the grouping variables incubation stand and litter mixture (different letters indicate significant differences, *p* < 0.05; *a* represents the lowest mean)

### Nutrient release

**Fig. 3:**
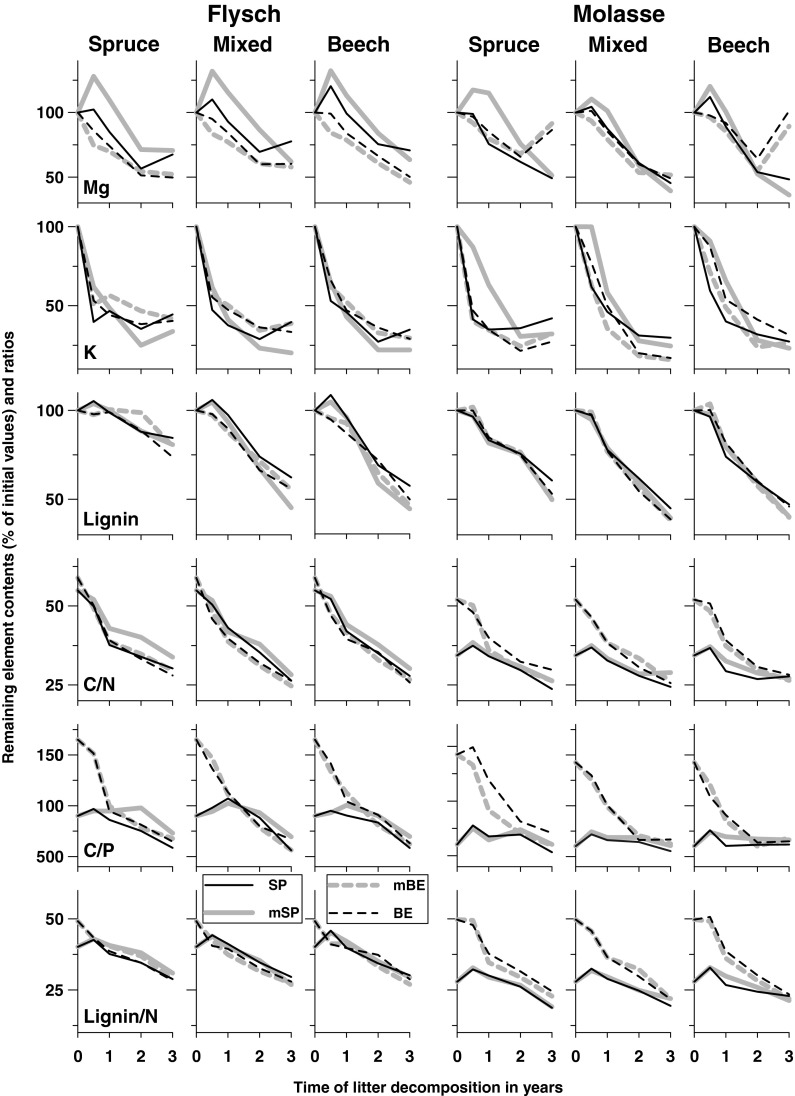
Mean remaining contents of Mg, K and lignin (percent of initial values) and changes of C_org_/N_tot_, C_org_/P and lignin/N ratios of exposed litter mixtures in single spruce (SP), mixed (mixed spruce, mSP; mixed beech, mBE) and single beech (BE) litter bags after 0.5, 1, 2 and 3 years, grouped by bedrock (Flysch, Molasse) and incubation stand (spruce, mixed, beech)

Remaining carbon contents showed the same patterns as the remaining masses, but mass loss did not correspond to nutrient release patterns. As reported elsewhere (*e.g.*, Prescott *et al*. 1993; Albers *et al*. 2004) nutrient immobilization during the early phases of decomposition followed by release of the same nutrient during later phases was visible in beech litter (mBE, BE) for N_tot_, P and S (Fig. 2) and in spruce litter (SP, mSP) for Mg (Fig. 3). On Flysch, there was a trend for part of the immobilized N_tot_, P and S in mixed beech litter to be transferred from mixed spruce litter within the same bags (since the differences between the single and mixed litter were roughly the same for both species: SP − mSP = mBE − BE; Fig. 2), however, Mg was transferred from mixed beech litter to mixed spruce litter (BE − mBE = mSP − SP; Fig. 3). On Molasse, transfer from one species to the other species was not observed as remaining element contents in single and mixed litter were similar.

The strong increase in the remaining contents of Ca (Fig. 2), Mg (to a lesser extent; Fig. 3) and Mn (not shown) in beech litter on Molasse at the last sampling date was possibly caused by external fungal Ca, Mg and Mn (Zeller *et al*. 2000), since fungi dominate the microbial community in moder and mor soils (Albers *et al*. 2004). In fact, after 3 years, beech litter on Molasse was to some extent visibly invaded by white-rot species, which are adapted to removal of lignin and other recalcitrant substances in litter previously partly decomposed by phyllosphere fungi (Osono 2003).

While the factors bedrock (soil type) and litter mixture had little effect on mass loss and C_org_ release, they explained much of the variation in nutrient release (immobilization) according to given *F*-values in Table 4. Litter mixture affected primarily the remaining contents of N_tot_, P and S and was the only factor controlling base cation release (other than time; Table 4). However, these differences (grouping variable litter mixture) after 3 years (Table 5) were mostly measured between leaves and needles in general and were not affected by mixing effects within the beech litter compounds (mBE = BE). Differences between single spruce and mixed spruce litter were significant for the remaining contents of S, K and lignin (mSP < SP) and for the ratios C_org_/N_tot_ and C_org_/P (mSP > SP), indicating faster decomposition of spruce litter in the mixed bags. This argument is supported by the highest mean C_mic_/C_org_ ratio in mixed spruce litter (mSP > mBE, BE; mSP = SP; SP = mBE, BE; Table 5).

Higher microbial N_tot_ and P immobilization rates of beech litter, indicated by a negative net N_tot_ and P release of beech litter under spruce (Table 6), resulted in a characteristic converging trend in C_org_/N_tot_, C_org_/P and lignin/N_tot_ ratios for all litter compounds and incubation stands on both bedrocks during decomposition (Fig. 3). The observed net immobilization of Al and Fe (remaining element contents above 100% in Table 5; negative net releases in mg g^−1^ litter in Table 6) was in accordance with Schlesinger (1997), reporting that plant litter appears to absorb Al and Fe, perhaps in compounds that are precursors to the fulvic acids. Net immobilization of Al, Fe and Mn (see above) was higher in beech than in spruce litter (see Tables 5, 6). In addition, we observed a net increase in remaining Na contents in spruce litter (Table 5).

**Table 6:** Net nutrient release (mg g^−1^ litter) of exposed litter mixtures in single spruce (SP), mixed (mixed spruce, mSP; mixed beech, mBE) and single beech (BE) litter bags over 3 years (Dec 2004–Nov 2007) averaged over both bedrocks by incubation stand (tree species composition: spruce, mixed beech-spruce, beech). Results of linear regressions (including a constant) between initial nutrient contents (mg g^−1^; independent variable) and net nutrient release (mg g^−1^ litter; dependent variable) are given if significant: determination (*r²*) and slope (*m*)

Element	Parameter	Spruce				Mixed				Beech			
		SP	mSP	mBE	BE	SP	mSP	mBE	BE	SP	mSP	mBE	BE
C_org_	Release	174.3	192.9	194.1	194.5	280.6	303.8	289.5	284.4	279.3	306.2	290.7	277.3
	*r²*			0.64 ^(^*^)^									
	*m*			3.58									
N_tot_	Release	0.25 *ab*	2.11 *b*	-1.85 *a*	-1.58 *a*	3.74 *ab*	5.61 *b*	0.79 *a*	0.84 *a*	4.47 *b*	5.56 *b*	1.21 *a*	0.96 *a*
	*r²*	0.68 *	0.84 *	0.64 ^(^*^)^		0.87 **	0.93 **	0.57 ^(^*^)^	0.82 *	0.74 *	0.80 *		
	*m*	0.52	0.65	0.93		0.68	0.78	0.69	0.92	0.60	0.57		
P	Release	0.13 *b*	0.25 *b*	-0.12 *a*	-0.09 *a*	0.32 *b*	0.41 *b*	0.00 *a*	0.03 *a*	0.35 *b*	0.42 *b*	0.05 *a*	0.01 *a*
	*r²*	0.90 **	0.91 **	0.88 **	0.85 **	0.92 **	0.93 **	0.90 **	0.88 **	0.89 **	0.92 **	0.88 **	0.60 ^(^*^)^
	*m*	0.82	0.76	0.78	0.82	0.80	0.75	0.88	0.92	0.75	0.68	0.97	0.68
S	Release	0.06	0.23	0.07	0.08	0.37	0.50	0.33	0.31	0.42	0.52	0.38	0.31
	*r²*	0.65 ^(^*^)^	0.74 *			0.77 *	0.91 **			0.71 *	0.87 **		
	*m*	0.76	0.86			0.71	0.88			0.65	0.72		
Ca	Release	3.26	2.38	1.91	1.51	2.56	2.88	3.14	2.89	2.55	2.85	1.67	1.16
	*r²*	0.98 ***	0.89 **	0.84 *	0.83 *	0.62 ^(^*^)^	0.88 **	0.71 *	0.61 ^(^*^)^	0.83 *	0.90 **	0.78 *	0.78 *
	*m*	0.50	0.28	0.89	0.83	0.28	0.32	0.58	0.51	0.32	0.25	0.79	0.91
Mg	Release	0.23	0.22	0.37	0.41	0.21	0.27	0.48	0.46	0.22	0.27	0.44	0.37
	*r²*			0.76 *	0.86 **			0.64 ^(^*^)^				0.87 **	0.82 *
	*m*			0.85	0.94			0.61				1.06	1.11
K	Release	0.86	1.02	1.58	1.68	0.99	1.20	1.84	1.89	1.05	1.19	1.81	1.74
	*r²*	0.78 *	0.90 **	0.67 *	0.82 *	0.63 ^(^*^)^	0.94 **	0.72 *	0.76 *	0.83 *	0.96 **	0.82 *	0.75 *
	*m*	0.53	0.70	0.82	0.96	0.69	0.95	1.13	1.05	0.74	0.79	0.98	0.84
Na	Release	-0.05	0.00	0.06	0.08	0.00	0.01	0.08	0.07	0.02	0.03	0.10	0.06
	*r²*		0.90 **	0.95 ***	0.86 **	0.91 **	0.88 **	0.96 ***	0.89 **	0.91 **	0.97 ***	0.88 **	
	*m*		1.20	1.22	1.03	1.02	0.96	0.98	1.08	0.98	1.07	0.70	
Al	Release	-0.33 *ab*	0.06 *b*	-1.38 *a*	-1.32 *a*	-0.41	0.31	-1.03	-0.83	-0.26 *ab*	0.23 *b*	-0.87 *a*	-1.18 *a*
	*r²*		0.56 ^(^*^)^				0.85 **			0.59 ^(^*^)^	0.79 *		
	*m*		0.74				1.07			1.07	0.67		
Fe	Release	-0.45 *ab*	-0.02 *b*	-1.06 *ab*	-1.59 *a*	-0.36	0.24	-0.71	-0.63	-0.26 *ab*	0.19 *b*	-0.59 *ab*	-0.89 *a*
	*r²*		0.56 ^(^*^)^		0.63 ^(^*^)^		0.86 **			0.62 ^(^*^)^	0.81 *	0.81 *	0.78 *
	*m*		0.83		37.76		1.08			1.14	0.71	-11.30	-17.41
Mn	Release	0.24 *b*	0.23 *b*	-0.51 *a*	-0.50 *a*	0.32 *ab*	0.45 *b*	-0.11 *a*	0.04 *ab*	0.29	0.46	-0.23	-0.33
	*r²*		0.79 *			0.84 **	0.98 ***			0.76 *	0.96 ***		
	*m*		0.26			0.34	0.58			0.41	0.53		
Lignin	Release	111.47	140.38	166.10	169.99	186.91	229.08	244.49	242.50	188.88	226.94	259.18	236.80
	*r²*		0.88 **	0.58 **		0.70 **	0.70 **		0.55 **		0.66 **		
	*m*		1.91	1.32		1.28	1.05		1.32		0.71		

A one-way ANOVA (factor litter mixture) was performed for each type of incubation stand over both bedrocks (*N* = 2 bedrocks × 3 replicated incubation stands × 4 litter mixtures × 1 stand mean of three replicated litter bags =24) and results of a Duncan multiple range test are given if net nutrient release values are significantly different (different letters indicate significant differences, *p* < 0.05; *a* represents the lowest mean). Regressions were done within each type of litter and incubation stand (*N* = 2 bedrocks × 3 replicated incubation stands × 1 litter mixture × 1 stand mean of three replicated litter bags =6). Level of significance of determination of regression (*r²*) is shown as: ^(^*^)^: *p* < 0.10; *: *p* < 0.05; **: *p* < 0.01; ***: *p* < 0.001

### Regression and correlation analyses

Net nutrient release (mg g^−1^ litter) over 3 years was regressed against initial nutrient contents for each incubation stand (grouped by litter mixture) over both bedrocks (soil type) in Table 6. Although the different soil types were hardly reflected in initial litter chemistry (see above, Table 2) the range of the data was large enough to yield positive relations (except for Fe, which were partially negative). We expected a negative relation for C_org_ indicating that increasing C_org_ litter contents decrease decay rates, but not a single correlation was significant at the level *p* < 0.05. High litter lignin contents increased lignin release (slopes between 0.71 and 1.91), questioning the recalcitrance of lignin and related compounds. The slopes (if significant) of all other nutrients (except Fe) were positive and within a quite narrow range (0.25–1.22) and did not differ significantly for a given element. For those elements showing retention (immobilization), regressions were not significant in most cases (N_tot_, S, Al, Fe, Mn in beech litter; Mg in spruce litter; see above). However, retention (immobilization) of P decreased significantly with increasing P litter contents.

We performed bivariate correlations between *k* and net nutrient release of exposed spruce and beech litter in single (SP and BE) and mixed (mSP and mBE) bags over 3 years, respectively, and numerous measured parameters according to Table 7. In a second step, those pre-selected parameters which correlated significantly with *k* or net release of the individual nutrients were used to run stepwise regressions to find the driving forces of *k* and release, yielding a model with the best suite of characteristics that control decomposition (Table 7). Relatively few parameters of litter, forest floor, mineral soil (0–10 cm) or the top soil (forest floor + mineral soil) were correlated with *k* and the parameter which was most useful in the model for characterizing *k* was “Beech” (note that the variables in the model are ranked by the corresponding partial regression coefficients in decreasing order). The fact that both models (SP and BE *versus* mSP and mBE) were similar (the same variables were kept via stepwise regression) indicates that mixture effects on decay rates were minimal. “Beech” stands for admixture of beech, representing an environmental parameter, which can not be contributed to a single horizon and is considered important in case features of the soil environment are not encompassed by the manifold soil chemical parameters. Hence, this kind of data analysis is in accordance with previous results (see above) stating that *k* was mainly controlled by the incubation stand type and mixing effects were not measured within leaf (mBE = BE) or needle (SP = mSP) litter (Table 5).

**Table 7:** Bivariate correlations between *k* (year^−1^) and net nutrient release (mg g^−1^ litter) of exposed spruce and beech litter in single (SP and BE) and mixed (mSP and mBE) bags over 3 years, respectively, and initial nutrient contents and element ratios of litter according to Table 3, as well as soil parameters of the forest floor (floor), 0–10 cm mineral soil (min. soil) and top soil (floor + min. soil) according to Table 2. The following parameters, not listed in Table 2 were included: sum of base cations (Base cat. = Ca+Mg+K+Na in mmol_c_ m^−2^ horizon^−1^), soil pH (CaCl_2_; min. soil) and mean volumetric water content (H_2_O; %; min. soil). Further correlations were done with the following parameters which can not be contributed to a single horizon but to all horizons (all) jointly: *k* and admixture of beech (Beech; dummy variable: 0 = spruce stand, 50 = mixed stand, 100 = beech stand). Only significant correlations, ranked in decreasing order, are given in bold (*p* < 0.001), italic (*p* < 0.01) and normal (*p* < 0.05) letters for relevant horizons (*N* = 2 bedrocks × 3 incubation stands × 3 replications; sites × 2 litter mixtures × 1 stand mean of three replicated litter bags =36). In a second step, these selected parameters without *k* were used to run stepwise regressions to select the driving forces (independent variables) of *k* and release. Model results of these multiple linear regression equations are shown; units are given in the captions of the cited tables; significance of adjusted coefficients of determination (*r* ^*2*^): ***: *p* < 0.001

*k*, Release	Horizon, Model	SP and BE Bivariate correlations (horizon), stepwise correlations (model)	mSP and mBE Bivariate correlations (horizon), stepwise correlations (model)
*k*	Litter	-Na	-Na
	Floor	Base sat.	Base sat.
	Min. soil	*Mg, C*_*mic*_ */C*_*org*_ *,* C_org_/N_tot_, -N_tot_, K	*C*_*mic*_ */C*_*org*_ *, Mg,* C_org_/N_tot_
	Top soil	C_org_/N_tot_, Ca, C_mic_/C_org_	C_org_/N_tot_, Base sat., Ca, C_mic_/C_org_
	All	*Beech*	**Beech**
	Model	*k* = 0.0333 + 0.0010 Beech − 0.4105 Litter Na + 0.0076 Top C_org_/N_tot_ + 0.0008 Top Ca; *r²* = 0.67^***^	*k* = 0.0602 + 0.0013 Beech − 0.4360 Litter Na + 0.0069 Top C_org_/N_tot_ + 0.0007 Top Ca; *r²* = 0.68^***^
C_org_	Floor	*Base sat.*	*Base sat.,* C_org_/P
	Min. soil	**C**_**mic**_ **/C**_**org**_, Mg, Base sat., −N_tot_, Base cat., Ca	*C*_*mic*_ */C*_*org*_, −N_tot_
	Top soil	**C**_**mic**_ **/C**_**org**_, Base sat., Ca, -N_tot_	Base sat., C_mic_/C_org_, Ca
	All	***k***, **Beech**	***k***, **Beech**
	Model	Release = 265.1036 + 0.8917 Beech − 0.4064 Min N_tot_; *r²* = 0.41^***^	Release = 269.5344 + 1.0058 Beech − 0.3777 Min N_tot_; *r²* = 0.51^***^
N_tot_	Litter	**P**, **-C**_**org**_ **/N**_**tot**_, **-Lignin, N**_**tot**_, **-C**_**org**_ **/P**, **S**, *Fe, Al,* -Ca	**P**, **-Lignin**, **-C**_**org**_ **/N**_**tot**_, **N**_**tot**_, **-C**_**org**_ **/P**, *S, Fe, Al,* -Ca, -Mg, -K
	Min. soil	*C*_*mic*_ *, C*_*org*_ */N*_*tot*_, Fe, S, P	*C*_*mic*_ *, C*_*org*_ */N*_*tot*_
	Top soil	*C*_*mic*_ *,* C_org_/N_tot_, P, Base cat., S	*C*_*mic*_ *,* C_org_/N_tot_, P, S, Base cat.
	All	***k*** *, Beech*	***k***, Beech
	Model	Release = 1.1296 + 5.8570 Litter P + 0.0338 Beech − 0.0912 Litter C_org_/N_tot_; *r²* = 0.80^***^	Release = − 6.3614 + 12.8134 Litter P + 0.0326 Beech; *r²* = 0.84^***^
P	Litter	**P**, **-Lignin/N**_**tot**_, **-C**_**org**_ **/P**, **-C**_**org**_ **/N**_**tot**_, **N**_**tot**_, *S, Al, Fe*, -C_mic,_ -C_mic_/C_org_, -Ca, -K	**P**, **-Lignin/N**_**tot**_, **-C**_**org**_ **/P**, **-C**_**org**_ **/N**_**tot**_, **N**_**tot**_, *Al, Fe*, S, *-C*_*mic*_ *, -C*_*mic*_ */C*_*org*_, -K, -Mg, -Ca
	Min. soil	C_mic_, C_org_/N_tot_	C_mic_, C_org_/N_tot_
	Top soil	C_mic_, Base cat.	C_mic_
	All	***k***	***k***
	Model	Release = − 0.3191 + 0.8193 Litter P; *r²* = 0.80^***^	Release = − 0.7858 + 1.2396 Litter P + 0.0056 Litter C_org_/N_tot_; *r²* = 0.86^***^
S	Litter	**-C**_**org**_ **/N**_**tot**_ *, S, N*_*tot*_ *, P, -Lignin/N*_*tot*_, -C_org_/P, Fe, Al	**-C**_**org**_ **/N**_**tot**_, **N**_**tot**_, **P**, **-Lignin/N**_**tot**_ **, S** *, -C*_*org*_ */P, Fe, Al*, Ca
	Min. soil	*C*_*mic*_ *,* C_org_/N_tot_, C_mic_/C_org_, Mg	*C*_*mic*_ *, C*_*org*_ */N*_*tot*_
	Top soil	C_mic_, C_org_/N_tot_, Base cat., Ca	*C*_*mic*_ *, C*_*org*_ */N*_*tot*_, P
	All	***k*** *, Beech*	***k*** *, Beech*
	Model	Release = 0.5311−0.0102 Litter C_org_/N_tot_ + 0.0026 Beech + 0.0023 Top Ca; *r²* = 0.63^***^	Release = 0.4808−0.0111 Litter C_org_/N_tot_ + 0.0029 Beech + 0.0121 Top C_org_/N_tot_; *r²* = 0.76^***^
Ca	Litter	**Ca**, **-C**_**org**_, **-Lignin** *, Mg*	**Ca**, **-C**_**org**_, **Mg**, *-Lignin*
	Floor	**-N**_**tot**_, **-S**, **-C**_**org**_, **-P**, **-C**_**mic**_, **-Na**, **-Al**, **-Acid cat.**, **-CEC**, **-Fe**, **Base sat.**, **-Base cat.**, **-K**, *-Mg, -Ca*, C_org_/N_tot_	**-N**_**tot**_, **-S**, **-C**_**org**_, **-P**, **-Na**, **-C**_**mic**_, **-Al**, **-Acid cat.**, **-CEC**, **-Fe**, **-Base cat.**, **Base sat.**, **-K**, *-Mg, -Ca*, C_org_/N_tot_, C_mic_/C_org_
	Min. soil	**-C**_**org**_, **H**_**2**_ **O**, *Ca, pH, Base cat., Base sat., -C*_*org*_ */P, -S, Mn, Mg, C*_*mic*_ */C*_*org*_, CEC, Na, -Fe	*pH* ***,*** *Ca, Base cat., H*_*2*_ *O, -C*_*org*_ *, -C*_*org*_ */P, Base sat., C*_*mic*_ */C*_*org*_ *, CEC, Mg,* Mn, Na, -S, K
	Top soil	**-C**_**org**_, **-N**_**tot**_, **-S**, **-C**_**mic**_, **-Al**, **-Acid cat.**, **-CEC**, **-Fe**, **-C**_**org**_ **/P**, *-P, Base sat., C*_*mic*_ */C*_*org*_ *, -K, -Mg*, Ca	**-C**_**org**_, **-N**_**tot**_, **-S**, **-Acid cat.**, **-Al**, **-CEC**, **-Fe**, **-C**_**mic**_, **-C**_**org**_ **/P**, *C*_*mic*_ */C*_*org*_ *, Base sat., -Mg, -K, -P*, Ca
	Model	Release = 9.0604−0.0012 Top C_org_; *r²* = 0.55^***^	Release = 8.0959 + 0.4831 Litter Ca − 0.0233 Litter Lignin; *r²* = 0.65^***^
Mg	Litter	**Mg**, **Ca**, K, C_mic_/C_org_, C_mic_	**Mg**, **Ca**, C_mic_/C_org_, C_mic_, K
	Floor	-N_tot_, -S, C_mic_/C_org_, -C_org_, -P, -Na	*-N*_*tot*_ *, -S, -Na, -C*_*org*_ *, -P*, -Al, -Acid cat., -CEC, -Fe, -C_mic_, -K, -Base cat., C_mic_/C_org_
	Min. soil	*Mg, Mn*, Ca, Base cat., -C_org_, Base sat., C_mic_/C_org_	*Mg, Ca, Base cat., Base sat.*, Mn, C_mic_/C_org_, pH, -C_org_
	Top soil	*-N*_*tot*_ *, -C*_*org*_, -S, Ca, -Na, Mn, C_mic_/C_org_, -Al, Base sat., -Acid cat.	*-N*_*tot*_ *, -C*_*org*_ *, Ca, Base sat., C*_*mic*_ */C*_*org*_, -Al, -C_org_/P, -S, -Acid cat., -Fe, -CEC
	All	k	–
	Model	Release = − 0.3978 + 1.1709 Litter Mg − 0.2277 Litter K − 0.0347 Litter Ca − 0.0022 Top N_tot_ + 0.0277 Top Mn − 0.001 Floor C_org_; *r²* = 0.79^***^	Release = − 0.4444 + 1.2871 Litter Mg − 0.2301 Litter K − 0.0438 Litter Ca − 0.0011 Top N_tot_; *r²* = 0.77^***^
K	Litter	**K**, **Mg**, *C*_*mic*_ */C*_*org*_ *, C*_*mic*_, Lignin	**K**, **Mg**, *C*_*mic*_ */C*_*org*_ *, C*_*mic*_
	Min. soil	*Mg*, K, Base cat., Ca, C_org_/N_tot_, Base sat.	**Mg**, *K, Base cat., Ca*, Base sat., Mn, C_mic_/C_org_
	Top soil	*Ca*, C_org_/N_tot_, Base cat.	*Ca*, Base sat., Base cat., C_mic_/C_org_
	Model	Release = − 0.9336 + 0.8030 Litter K + 0.0318 Top C_org_/N_tot_; *r²* = 0.88^***^	Release = − 0.1251 + 0.7821 Litter K; *r²* = 0.78^***^
Na	Litter	**Na**, *-Mn*, Lignin	**Na**, *Lignin, -Mn*
	Top soil	Na	*Na*
	Model	Release = − 0.1061 + 0.9778 Litter Na; *r²* = 0.70^***^	Release = − 0.1007 + 1.0519 Litter Na; *r²* = 0.89^***^
Lignin	Litter	**Lignin** *, C*_*org*_ *, -Ca,* C_mic_, C_mic_/C_org_, -C_org_/N_tot_	**Lignin** *, -Ca,* -C_org_/N_tot_, Corg, N_tot_
	Floor	*Ca, C*_*mic*_ *, N*_*tot*_ *, C*_*org*_ *, S, P, Na, Al, CEC, Acid cat., Base cat*, Fe, K	*Ca, N*_*tot*_ *, C*_*org*_ *, C*_*mic*_ *, S, P, Na*, Al, Acid cat., CEC, Base cat, Fe
	Min. soil	*C*_*org*_ */N*_*tot*_ *, Fe*, C_mic_, -H_2_O, -Na, -pH	*C*_*org*_ */N*_*tot*_ *, C*_*mic*_ *, Fe*, -H_2_O, C_org_
	Top soil	***C***_***mic***_, ***C***_***org***_ ***/N***_***tot***_, *C*_*org*_ *, S, CEC,* Al, Acid cat., Fe, K, P, C_org_/P, N_tot_, Mg, Base cat.	***C***_***mic***_, ***C***_***org***_ ***/N***_***tot***_, *Corg, S,* P, CEC, C_org_/P, Al, Acid cat., N_tot_, Fe, K
	All	***k***, Beech	***k*** *, Beech*
	Model	Release = − 261.0391 + 0.8166 Litter Lignin + 0.7233 Beech + 28.2838 Top C_org_/N_tot_ + 4.9571 Floor Ca − 22.7114 Min C_org_/N_tot_ + 302.0128 Min Fe − 26.0829 Floor Na − 4.9567 Min H_2_O; *r²* = 0.85^***^	Release = − 274.4414 + 1.0605 Beech + 1.6337 Floor Ca + 25.2003 Top C_org_/N_tot_ + 0.4849 Litter Lignin − 17.8544 Min C_org_/N_tot_ + 204.2422 Min Fe; *r²* = 0.81^***^

It is striking that *k* and nutrient release were positively related with the C_org_/N_tot_ ratio of the forest floor or mineral soil but for many nutrients negatively with the litter C_org_/N_tot_ ratio (Table 7). Wide C_org_/N_tot_ ratios may stand for the fact that N-rich components are quickly decomposed and mineralized and do not necessarily point to retarded decomposition as usually cited in the literature. There are other examples in Table 7 that a specific parameter of the litter favored release while the same parameter of the forest floor or mineral soil hindered release of the same element. Whether a nutrient is released quickly or slowly depended on the specific horizon. For example, P release was primarily related to litter chemistry (having many correlations with litter, but few with forest floor or mineral soil parameters). In contrast, Ca release was mainly correlated with forest floor and mineral soil chemistry (with few correlations with litter parameters).

In general, differences between the model results using data of either lumped SP and BE or mSP and mBE litter were minimal, supporting above results that nutrient release from decomposing beech or spruce litter was quite similar when incubated in single species or mixed species bags (Table 7). Only for Ca were the variables kept in the two models different: Ca release in single species bags was primarily affected by soil chemistry (Top C_org_) and in mixed species bags mainly by litter chemistry (Litter Ca, Litter Lignin).

The initial element content in the litter explained most of the variation in the release of the same element for P, Mg, K, Na and lignin. The (non-chemical) soil environment (*e.g.*, micro-climate, physical conditions, activity of decomposing organisms), expressed by the variable “Beech” primarily controlled decomposition rate *k* (see above) and C_org_ release (listed as first variable) and to a lesser extent (listed at the second place or later) release of N_tot_, S and lignin. Keeping soil parameters in the model additionally improved *r²* of the stepwise regressions for S, Mg, K and lignin (Table 7).

## Discussion

### Question 1: Does beech litter decompose faster than spruce litter?

i) Mass loss of beech litter was not higher than mass loss of spruce litter. During the first year decay of beech litter was significantly lower than of spruce, but differences declined over time. ii) Net nutrient release (after 3 years) of N_tot_, P, Ca, Al, and Mn was higher in spruce than in beech litter due to high immobilization (retention) rates of beech litter. iii)However, beech litter released more Na than spruce litter.

Our (implicit) expectation that the broadleaf component decays faster was not fulfilled. Slower decay of beech *versus* spruce litter is in accordance with Vesterdal (1999; at one of 3 sites only), Albers *et al*. (2004) and Sariyildiz *et al*. (2005; comparison between *Fagus orientalis* and *Picea orientalis*). This research has demonstrated that the purported faster decomposition of beech leaf litter is not a safe generalization to make, and is obviously not the cause of the differences in soils beneath the two species.

### Question 2: Does litter decompose faster in beech or beech-spruce forests than in spruce forests?

i) Decay and C_org_ release were primarily affected by tree species composition of the incubation stand and were faster in (mixed) beech forests stands than in spruce forests. Litter decay indicated non-additive patterns, since similar remaining masses under pure beech (47%) and mixed beech-spruce (48%) were significantly lower than under pure spruce stands (67%). The same patterns were found for C_org_, N_tot_, P, S and lignin, compounds building up the organic litter layer, and associated K. ii) However, release of all other nutrients (Ca, Mg, Na, Al, Fe, Mn) was not affected by stand tree species composition.

Though beech litter itself did not decay faster than spruce litter, favorable environmental conditions in (mixed) beech stands increased litter decay. It is of practical relevance to know that the formation of thick organic layers in spruce monocultures, suggested to hamper productivity, can be avoided by admixture of beech and does not necessarily require complete stand conversion to pure beech. The fact that analyses of *k,* remaining mass and element contents using an ANOVA (Table 4) did not show a single significant interaction “incubation stand x litter mixture” indicates that home-field advantage (HFA; *i.e.*, faster decomposition of litter at home than away) was not useful for explaining variation of litter decomposition in this study. Nevertheless, we used our reciprocal litter transplant experiment, where leaf litter from spruce and beech were decomposed over 3 years (last sampling date only) at pure spruce and pure beech stands to calculate HFA for each bedrock according to Ayres *et al*. (2009). For pairwise comparisons of tree species, the HFA index gives the percentage of a more rapid (positive value) or slower (negative value) mass loss of litter when it decomposes under the tree species from which it had been derived (*i.e.*, “at home”). Calculated mean HFA was negligible on Flysch (+0.4) but negative on Molasse (−6.2; data in % faster decomposition at home). As visible in Fig. 2, the negative HFA index on Molasse was caused by the fact that mass loss under the beech stand was higher for spruce than for beech litter. A negative HFA index is rare (77% of 35 reciprocal leaf litter transplants exhibited a net stimulation of decomposition at home with a mean HFA of +8.0%; Ayres *et al*. 2009) but not unusual when beech litter is involved: pairwise comparisons of the decomposition of beech litter with litter of other broadleaf species (*k* was slowest in beech litter) revealed a negative mean HFI index in the short term (1 and 4 months) but a home field advantage (positive index) after 7 months (Jacob *et al*. 2010).

### Question 3: Does mixing of beech and spruce litter hasten decomposition of spruce litter?

i) Mass loss did not differ between single and mixed spruce litter or between single and mixed beech litter. ii) Mixing beech and spruce litter tended to increase decay of spruce needles after the first year. iii) Nutrient release was not affected by mixing effects within the beech litter compounds (mBE = BE; Table 5). iv) In a few cases, remaining contents (S, K, lignin) and final litter ratios (C_org_/N_tot_ and C_org_/P) were different between single spruce and mixed spruce litter, indicating somewhat faster decomposition of spruce litter in the mixed bags.

It is hypothesized that enhanced decay rate and nutrient release in mixtures of litter, as shown by a number of authors (Gartner and Cardon 2004 and references therein), is caused by translocation of nutrients between litters of different quality, resulting in a more rapid and efficient utilization of litter substrate by decomposers. Net transfers of nutrients between the two litter species in the mixed bags were minimal. However, differences of initial litter chemistry between beech and spruce were negligible or indicated even lower quality of beech litter (with the exception of higher base cation contents in beech litter).

### Question 4: Does mass loss (decay rate) correlate with nutrient release?

i) Remaining carbon and lignin contents in decomposing litter showed the same temporal patterns as the remaining masses. ii) Despite immobilization (retention) during early phases of decomposition for N_tot_, P and S (beech litter) the corresponding element contents correlated with the remaining masses, 3 years after the start of the experiment. iii) Nutrient release of base cations (except K), Mn and Fe was not related to mass loss.

The significant bivariate correlations above (performed for SB and BE, in each case: *N* = 18) were documented by the following positive coefficients (*R*) between the remaining masses and the remaining element contents after 3 years in decreasing order: C_org_: 0.94^***^, 0.96^***^; lignin: 0.83^***^, 0.87^***^; S: 0.73^**^, 0.85^***^; N_tot_: 0.67^**^, 0.79^***^; P: 0.70^**^, 0.69^**^; K: 0.71^**^; 0.69^**^ (SP *vs*. BE; ^***^: *p* < 0.001; ^**^: *p* < 0.01). Hence, mass loss was driving the release of the main components of the organic substance and associated K. Berger *et al*. (2009b) estimated mean residence times (T_n_ = forest floor content divided by annual inputs) of individual nutrients within the forest floor for one of the same sites on Flysch and one of the same sites on Molasse, which decreased in all cases from spruce over the mixed to the beech stands according to accelerated litter decay with increasing admixture of beech (see question 2). Because individual residence times are quite different, we can not expect that nutrient release is solely determined by mass loss, as our data have shown. In agreement with much higher mean C_org_ contents of the forest floor on Molasse than on Flysch (Table 2), T_n_ is higher on that site on Molasse as well (*e.g.*, T_n_ for S, P and N_tot_ is 8–12 years on Molasse *vs*. 1–6 years on Flysch for the mixed stands; Berger *et al*. 2009b). The litter bag method did not reveal similar bedrock effects at all. Within 3 years, net release of N_tot_, P, S was even higher on Molasse (Table 5). There are two possible explanations: a) three years of decomposition still encompasses early phases of decomposition and no conclusions about steady (final) state conditions are justified. b) On Molasse, initial organic litter compounds are quickly decomposed to soluble low molecular weight organic compounds (acids) and leached out of the litter bag to the deeper horizons of the forest floor. Hence, disappearance from the litter bags does not mean complete but incomplete decomposition of organic compounds and transfer within thick humus layers.

### Question 5: Which parameters (litter, soil, environment) represent the best suite of characteristics that actually control decay rates and nutrient release?

i) Plant-induced changes in the soil environment (*e.g.*, micro-climate, physical conditions, activity of decomposing organisms; expressed by a dummy variable for increasing admixture of beech from spruce over mixed to beech stand), not encompassed by the measured soil chemical parameters, primarily controlled *k*, C_org_ release and to a lesser extent release of N_tot_, S and lignin. ii) The initial element content in the litter explained most of the variation in the release of the same element for P, Mg, K, Na and lignin. iii) Chemical soil parameters explained part of the remaining variance in release of S, Mg, K and lignin.

## Conclusions

In contradiction to the widely held assumption of slow decomposition of spruce needles (but in accordance with Albers *et al*. 2004) we conclude that accumulation of litter in spruce stands is not caused by recalcitrance of spruce needles to decay; rather adverse environmental conditions in spruce stands retard decomposition. Mixed beech-spruce stands appear to be as effective as pure beech stands in counteracting these adverse conditions, preventing the accumulation of thick organic layers observed in spruce monocultures.
